# Improving Minimum Cross-Entropy Thresholding for Segmentation of Infected Foregrounds in Medical Images Based on Mean Filters Approaches

**DOI:** 10.1155/2022/9289574

**Published:** 2022-03-17

**Authors:** Walaa Ali H. Jumiawi, Ali El-Zaart

**Affiliations:** Department of Mathematics and Computer Science, Faculty of Science, Beirut Arab University, Beirut, Lebanon

## Abstract

Mean-based thresholding methods are among the most popular techniques that are used for images segmentation. Thresholding is a fundamental process for many applications since it provides a good degree of intensity separation of given images. Minimum cross-entropy thresholding (MCET) is one of the widely used mean-based methods for images segmentation; it is based on a classical mean that remains steady and limited value. In this paper, to improve the efficiency of MCET, dedicated mean estimation approaches are proposed to be used with MCET, instead of using the classical mean. The proposed mean estimation approaches, for example, alpha trim, harmonic, contraharmonic, and geometric, tend to exclude the negative impact of the undesired parts from the mean computation process, such as noises, local outliers, and gray intensity levels, and then provide an improvement for the thresholding process that can reflect good segmentation results. The proposed technique adds a profound impact on accurate images segmentation. It can be extended to other applications in object detection. Three data sets of medical images were applied for segmentation in this paper, including magnetic resonance imaging (MRI) Alzheimer's, MRI brain tumor, and skin lesion. The unsupervised and supervised evaluations were used to conduct the efficiency of the proposed method.

## 1. Introduction

Thresholding is a type of image segmentation that tends to find a good point to separate heterogeneous regions or group objects and split them from the background [1]. The optimal threshold is the value that indicates the degree of intensity separation for a given image, for example, in medical images, that either indicates extreme diseases, or vice versa. To analyze the features and segmented objects inside the image, there are two important factors in image intensities on which the segmentation process depends. The first factor is the change ratio in intensity levels and to what extent they are discontinuous, especially the edges of objects in an image. The second factor is to what extent the intensities are the same. These factors are applied to entropic thresholding [2–5]. Segmentation mainly depends on the threshold value to separate objects from the background. The value of the optimal threshold can be obtained from various methods. The mean-based minimum cross-entropy thresholding (MCET) is one of the frequently used for image segmentation, where various distributions are predicted using the image histogram [6, 7]. This paper aims to improve MCET, by estimating the desired mean values from the image regions. This contributes to computer-aided diagnosis (CAD) techniques by improving MCET for image segmentation that aids physicians' assessment of the degree of the disease.

Computer-aided diagnosis (CAD) systems have significant potential for diagnosing fatal diseases. Segmentation of infected foreground in medical images has an important role in detecting malignant disease, or vice versa [8]. Alzheimer's disease (AD) is a gradual neurologic disease in the brain that produces irreversible loss of neurons. AD leads to tissue loss throughout the brain that shrinks the size of brain volume. The number of affected people is increasing every decade; within the next two decades, 1 out of 85 persons will have the AD by 2050 [9, 10]. CAD systems can help neurologists discover the early stage of AD. Medical resonance imaging (MRI) has been confirmed to be very useful in this task [11]. Technically, the CAD system uses MRI to display a useful comparison between normal and abnormal neurons using the analysis of certain characteristics in brain images and thus determine the appearance of atrophied neurons [12, 13]. Image segmentation is an important technique that detects infected skin lesions and separates the lesion from the skin region. To provide reliable diagnosis, CAD systems tend to observe the early symmetric shape [8, 14]. Moreover, MRI brain tumor segmentation is a hard task for many reasons, such as the non-homogeneous intensities around the tumor, the presence of noises in the background, the complicated shapes and the fuzzy boundaries, and less contrast between neighboring brain tissues [15–17]. Standard segmentation methods of MRI brain tumors are time-consuming and difficult and provide different results. In this paper, the proposed model tends to improve the segmentation accuracy of infected foreground in the medical image for better diagnosing purposes. The main contributions of this research are as follows:

- Improving minimum cross-entropy thresholding using different mean filters approaches
- Developing an image segmentation model for accurate and early detection of infected foreground in medical images
- Handling the challenge of noise and outliers in images that negatively impact the mean value in the segmentation model
- Implementing inclusive segmentation algorithm for various medical images and intensity distributions

## 2. Related Works

Several studies have been proposed and developed in the literature for thresholding-based segmentation. Some studies produced satisfying outputs, and they were dedicated to a certain type of images [18]. Others rely on image histograms or local properties, for example, local mean value and the local gradient or the standard deviation. The most conjectural approach is global thresholding based on histogram components. Here, one threshold is assigned for the entire image [19]. Global thresholding considers the image to have a bimodal histogram; therefore, the object can be segmented and separated from the background, by comparing image pixels with a threshold value *T* [1–4]. Suppose an image *I*(*x*, *y*) is given with its corresponding histogram. The thresholded image is defined as *g*(*x*, *y*), as follows:

(1) $$\begin{array}{c} g\left(x,y\right)=\left\{\begin{array}{cc} 1 & \text{if}\;I\left(x,y\right)\;>T,\\ 0 & \text{if}\;I\left(x,y\right)\;\leq T, \end{array}\right. \end{array}$$

where *T* is the optimal thresholded, *g*(*x*, *y*) is the thresholded image based on the distributed intensities in *I*(*x*, *y*), and *I*(*x*, *y*) is the original image. The result is a binary image, where the pixels equal to 255 correspond to objects, and the pixels equal to 0 correspond to the background [3, 20]. There are various types of global thresholding methods, for example, Otsu 1979, proposed by Otsu [3], and its relevant approaches. This paper focuses on entropy thresholding methods and specifically improving minimum cross-entropy thresholding (MCET) because it relies on the mean values in its technique as shown in equation (4), while Otsu is based on the mean and the variance.

Entropy is the measure of information components in a probability distribution [21, 22]. Entropy in images processing could be used to measure the division of two classes and therefore separate the information in images into two regions by the threshold. There are different types of entropy thresholding methods, for example, maximum entropy thresholding that maximizes the sum of two class entropies, fuzzy entropic thresholding that minimizes the sum of the fuzzy membership, and MCET that minimizes the variance between two class entropies [7].

### 2.1. Minimum Cross-Entropy Thresholding (MCET)

Minimum cross-entropy-based thresholding (MCET) describes the optimum threshold by minimizing the variance between two class entropies [22]; thus, the result of optimal threshold *t*^*∗*^ is as follows:

(2) $$\begin{array}{c} t^{*}\min_{t}\left(D\left(I,I_{t}\right)\right)=\min_{t}\left(D\left(t\right)\right), \end{array}$$

where *D*(*I*, *I*_*t*_) can be written as *D*(*t*) and is determined as follows:

(3) $$\begin{array}{c} D\left(t\right)=\overset{L}{\underset{i=1}{\sum }}i*h\left(i\right)*\;\log\left(i\right)\;-\overset{L}{\underset{i=1}{\sum }}i*h\left(i\right)*\;\log\left(\mu_{1}\left(t\right)\right)\;-\overset{L}{\underset{i=t+1}{\sum }}i*h\left(i\right)*\;\log\left(\mu_{2}\left(t\right)\right), \end{array}$$

where *h*(*i*) refers to the histogram of the gray level *i* for the range [1, *L*] and *μ*_1_(*t*) and *μ*_2_(*t*) are the mean values of the first and the second region, respectively. Since the first term is a constant for a given image [8]; thus, the objective function can be written as follows:

(4) $$\begin{array}{c} n\left(t\right)=-\sum_{i=1}^{t}i*h\left(i\right)*\;\log\left(\mu_{1}\left(t\right)\right)-\sum_{i=t+1}^{L}i*h\left(i\right)*\;\log\left(\mu_{2}\left(t\right)\right). \end{array}$$

Therefore, this function can be rewritten as follows:

(5) $$\begin{array}{c} n\left(t\right)=C_{1}\left(t\right)*\;\log\left(\mu_{1}\left(t\right)\right)+C_{2}\left(t\right)*\;\log\left(\mu_{2}\left(t\right)\right), \end{array}$$

where *C*_1_(*t*) and *C*_2_(*t*) are as follows:

(6) $$\begin{array}{c} C_{1}\left(t\right)=-\sum_{i=1}^{t}i*h\left(i\right), \end{array}$$

(7) $$\begin{array}{c} C_{2}\left(t\right)=-\sum_{i=t+1}^{L}i*h\left(i\right). \end{array}$$

This method finds the best distribution for the image regions of an image based on the probabilistic distribution approach. The Gaussian distribution is not constantly suitable for symmetric classes in the image histogram due to the impact of noise and outliers. Mainly, the regions of the image are considered to be two Gaussian distributions, such that the value of *μ*_1_(*t*) and *μ*_2_(*t*) are estimated from the following equations:

(8) $$\begin{array}{c} \mu_{1}\left(t\right)=\frac{\;\sum_{i=1}^{t}i*h\left(i\right)}{\;\sum_{i=1}^{t}h\left(i\right)}, \end{array}$$

(9) $$\begin{array}{c} \mu_{2}\left(t\right)=\frac{\;\sum_{i=t+1}^{L}i*h\left(i\right)}{\;\sum_{i=t+1}^{L}h\left(i\right)}. \end{array}$$

In this mean-based method, the final threshold in equation (1) is computed depending on the mean value based on Gaussian distribution definition equations (8) and (9), to be applied on equation (4). The process of mean computation in MCET is similar to the approach of “arithmetic mean” regardless of whether the image histogram has a symmetric distribution or not. The presence of noise, local outliers, and gray areas will also be included in the mean computation and highly impact the calculation in equation (4). This case could produce drawbacks in regard to the final threshold especially due to the direct relationship between the mean value and the final threshold. This impact could lead to a negative effect on segmentation results.

Minimum cross-entropy thresholding was proposed under the Gaussian distribution for mean estimation [7, 22, 23]. The proposed methods showed some satisfying results with a limitation regarding the steady mean estimation formula that impacts the efficiency of the method function. The classical mean value usually includes all intensity levels and unwanted parts of image regions; thus, it could negatively impact the final threshold of the MCET. The method also improved for selecting multilevel threshold values using particle swarm optimization [21] and an improved human mental search algorithm [24]. Also, it was proposed for color image segmentation based on exchange market algorithm [25]. MCET also improved by using gamma, Gaussian distribution in [8]. Moreover, it was developed using hybrid distribution including Gaussian and lognormal [6]. Although the process of mean estimation remained steady for each selected distribution, the stated methods were able to achieve the satisfying performance of segmentation for a certain type of images, with some limitations regarding the computational range for the mean value.

**Figure 1:**
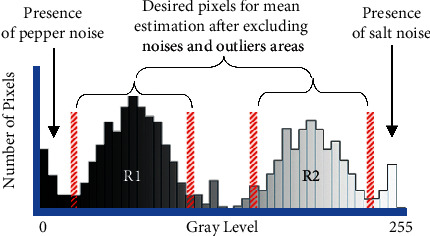
Histogram visualization for the proposed mean estimation techniques, excluding the unwanted areas for mean estimation, for example, alpha trim with specific *d*/2 trim value.

The original MCET is not always suitable for various types of images. Usually, Gaussian-based MCET is suitable for symmetric distribution. However, some images with symmetric distribution may face inaccurate segmentation issues because there can be a randomly distributed noise and local outliers inside each region in the image and gray intensity levels between the object and the background regions; thus, they can negatively impact the mean computation for the MCET as shown in Figure 1. These situations are some of the main challenges that can directly impact the desired threshold because their negative effects on mean values, as they are included in the classical mean computation process. MCET method completely relies on the mean values. Since the mean value is computed in a simple form, this value is not enhanced enough to be a much-desired mean value for this method. Thus, it computes a single value margin of the threshold. The stated drawbacks can directly affect the segmentation accuracy. One of the issues is how much the mean-based MCET is sensitive to noise and outliers that are included in the mean computation. This work ignores any process that tends to change the original pixels inside image regions. The goal is to find new enhanced mean values from these regions. Automatically selecting threshold from histogram gray-level values has been derived from the viewpoint of differential analysis including what is the desired mean for each region. This paper also tends to evolve MCET toward optimal thresholding technique by estimating new mean values using different approaches.

## 3. Proposed Mean Estimation Approaches

**Figure 2:**
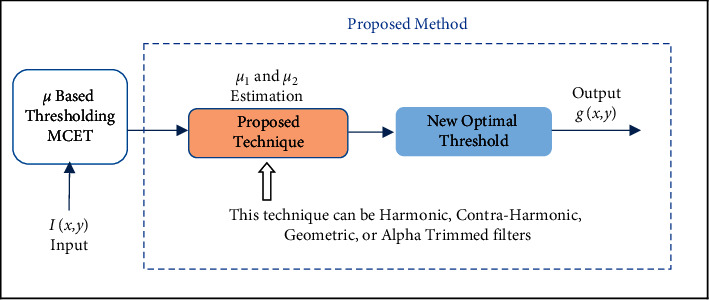
Proposed model, where *μ*_1_ and *μ*2 are estimated using mean filters approaches.

**Figure 3:**
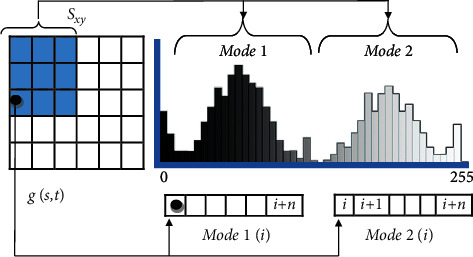
Modes adaptation for mean estimation filters, where the mask *S*_*xy*_ refers to the mask in the original filter, and the modes refer to vectors of pixels in histogram regions.

According to the stated issues in the introduction and related work, this paper proposes using existing mean estimation approaches depending on the image filtering techniques, to be used with histogram version for each class, and dealing with the pixels in each class assorted vectors. The proposed technique uses the mean filters for both *μ*_1_ and *μ*_2_ values in each class and then obtains a new optimal threshold as shown in Figure 2. Mean filters are adapted to be used in histogram version; for example, alpha trimmed in equation (10) is adapted in equations (11) and (12), which will eliminate the unwanted parts from histogram regions and exclude them with specific trim value, as shown in Figure 3.

In general, noise is distributed at the start and the end of the histogram, where pepper noise is present at the beginning of histogram gray level (the lowest gray level), and the salt noises is present at the end of the histogram (the highest gray level), as shown in Figure 1. Thus, the filtering approaches such as harmonic, contraharmonic, geometric, and alpha-trimmed mean filters [1] are used as mean estimators for MCET since the optimal threshold in this method is completely dependent on the classical mean value. From this point, the proposed techniques could provide a good indication of this relation. This indication aims to handle the drawbacks in the related work and improve the MCET method, as well as define clear formula for mean estimation, for various kinds of images and pixels distribution.

### 3.1. Classical Mean Filter

This filter is called the arithmetic mean filter and is the simplest mean filter, as shown in equation (10). It computes the average value of image *g*(*x*, *y*) in the area defined by *S*_*xy*_.

(10) $$\begin{array}{c} \hat{f}\;\left(x,y\right)=\frac{1}{mn}\;\sum_{\left(s,t\right)\in S_{xy}}g\left(s,t\right). \end{array}$$

The process of mean estimation in the histogram version is shown in the following equations:

(11) $$\begin{array}{c} \mu_{1}\left(t\right)\;=\frac{1}{\text{Length}1\;}\sum_{i\in \text{Mode}1}\text{Mode}1\left(i\right), \end{array}$$

(12) $$\begin{array}{c} \;\mu_{2}\left(t\right)\;=\frac{1}{\text{Length}2\;}\sum_{i\in \text{Mode}2}\text{Mode}2\left(i\right), \end{array}$$

where *μ*_1_(*t*) and *μ*_2_(*t*) are the mean values of mode1 (class1) and mode2 (class2) in the image histogram, respectively. *S*_*xy*_ refers to the mask in the original filter, and modes 1 and 2 refer to the vectors of pixels in histogram regions, as explained in Figure 3.

### 3.2. Alpha-Trimmed Mean Filter

This filter tends to enhance the mean value, by excluding noises and outliers parts from histogram regions by applying *α*-trimming value, as shown in equation (13). The intensity levels are treated as a sorted vector, while mean estimation in the histogram version is shown in equations (14) and (15). This filter can handle multiple types of noise in images, for example, combination of salt and pepper as well as Gaussian noise, as shown in Figure 1. This filter could provide a better mean value for asymmetric pixels distribution. Moreover, this filter could provide less affected error by outliers and asymmetries than using normal distribution when it is used with MCET for image segmentation.

(13) $$\begin{array}{c} \hat{f}\;\left(x,y\right)=\frac{1}{mn-d}\sum_{\left(s,t\right)\in S_{xy}}g_{r}\left(s,t\right), \end{array}$$

where *g*_*r*_(*s*, *t*) represent the remaining *m∗n* − *d* pixels after removing the *d*/2 highest and *d*/2 lowest values from the overall pixels in the region *g*(*s*, *t*) and *S*_*xy*_ represents the set of coordinates in a rectangular subimage or mask window of size *m∗n*.

**Figure 4:**
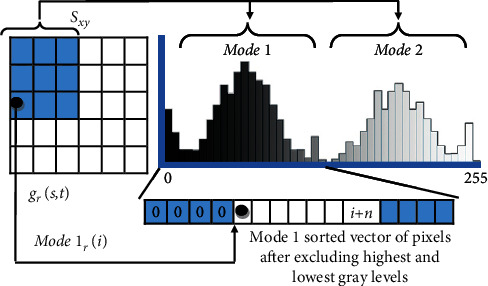
Modes adaptation for alpha-trimmed mean estimation, where the mask *S*_*xy*_ refers to the mask in the original filter, the modes refer to vectors of pixels in histogram regions, and *r* refers to the remaining pixels.

The formula of the alpha-trimmed filter can be rewritten for the histogram version as follows:

(14) $$\begin{array}{c} \mu_{1}\left(t\right)\;=\frac{1}{\text{Length}1\;-\;d}\sum_{i\in \text{Mode}1}{\text{Mode}1}_{r}\left(i\right), \end{array}$$

(15) $$\begin{array}{c} \;\mu_{2}\left(t\right)=\frac{1}{\text{Length }2-\;d}\sum_{i\in \text{Mode}2}\text{Mode}2_{r}\left(i\right), \end{array}$$

where Length1 and Length2 represent the lengths in the first and second regions or modes, respectively; Mode1 and Mode2 represent the overall pixels in each region that is similar to the approach of the mask *S*_*xy*_ in equation (13); and Mode1_*r*_(*i*) and Mode2_*r*_(*i*) represent the remaining pixels of after excluding the highest *d*/2 and the lowest *d*/2 trim values from vector in Mode1 and Mode2, respectively as shown in Figure 4. The modes are treated as sorted vectors, and the trim value should be applied on each mode, as it can be noticed that the grayness area represents the highest intensities when the filter is applied on the first mode, and it represents the lowest intensity when the trim is applied on Mode2, and the overall intensities that host the salt and pepper noises will remain the highest and lowest when the filter applied on Mode1 and Mode2.

The alpha-trimmed technique can be used for multiple trim values on both modes. In our proposed method, the same filter with its trim value should be used for *μ*_1_ and *μ*_2_ in each mode in the image histogram.

### 3.3. Harmonic Mean Filter

This filter tends to enhance the mean value, by excluding salt noise, but not for pepper noise, as shown in equation (16). It does work also with Gaussian noise; this could provide a good impact if the image has one of the stated noise.

(16) $$\begin{array}{c} \hat{f}\;\left(x,y\right)=\;\frac{mn}{\sum_{\left(s,t\right)\in S_{xy}}1/g\left(s,t\right)}. \end{array}$$

The harmonic technique in equation (17) is modified also to be compatible in histogram versions for mean estimation of a specific mode, where Length refers to the mode length or histogram region, and mode(*i*) represents the pixels of that mode, for example, mode 1 or mode 2, as shown in Figure 3.

(17) $$\begin{array}{c} \mu \left(t\right)=\;\frac{\text{Mode}\_\text{Length}}{\sum_{i\in \text{Mode}}1/\text{Mode}\left(i\right)}. \end{array}$$

### 3.4. Contraharmonic Mean Filter

This filter tends to enhance the mean value by eliminating the noises and outliers areas; this is for image enhancement purposes, as shown in equation (18). In this work, noise needs to be excluded from histogram modes for better mean estimation.

(18) $$\begin{array}{c} \hat{f}\;\left(x,y\right)=\frac{\sum_{\left(s,t\right)\in S_{xy}}g{\left(s,t\right)}^{Q+1}}{\sum_{\left(s,t\right)\in S_{xy}}g{\left(s,t\right)}^{Q}}. \end{array}$$

This filter in the histogram version can be written as follows:

(19) $$\begin{array}{c} \mu_{1}\left(t\right)\;=\frac{\sum_{i\in \text{Mode}1}\text{Mode}1{\left(i\right)}^{Q+1}}{\sum_{i\in \text{Mode}1}\text{Mode}1{\left(i\right)}^{Q}}, \end{array}$$

(20) $$\begin{array}{c} \mu_{2}\left(t\right)=\frac{\sum_{i\in \text{Mode}2}\text{Mode}2{\left(i\right)}^{Q+1}}{\sum_{i\in \text{Mode}2}\text{Mode}2{\left(i\right)}^{Q}}, \end{array}$$

where Mode1 and Mode2 represent the overall pixels for each class, respectively, and Mode1(*i*) and Mode2(*i*) are the vectors of pixels in each class, respectively, as shown in Figure 3. The *Q* value of the contraharmonic filter represents the order of the filter; this filter is used to exclude salt and pepper noises in the image, where positive *Q* excludes pepper noise and negative *Q* excludes salt noise, but not both noises, as well as contraharmonic reduces to the classical arithmetic mean if *Q* is equal to 0 and reduces to harmonic mean filter if *Q* is equal to −1. Changing the value of *Q* according to the proposed mean estimation technique can impact the desired threshold in MCET; hence, at some point, it tends to handle the drawbacks from the classical mean.

### 3.5. Geometric Mean Filter

Geometric mean filter tends to lose less image details in the image enhancement process, but this is not our case, while in segmentation, the aim is to focus on mean estimation for the thresholding method, as shown in equation (21). This filter restores the mean value by using product operation. This filter is proposed to compare its impact with the other filters. Knowing that the geometric mean filter achieves smoothing comparable to the arithmetic mean filter, which could provide less effect than the previously stated filters. This filter is adapted similarly to be used with histogram modes for mean estimation in equation (22).

(21) $$\begin{array}{c} \hat{f}\;\left(x,y\right)=\;{\left[\prod_{\left(s,t\right)\in S_{xy}}g\left(s,t\right)\right]}^{1/mn}, \end{array}$$

(22) $$\begin{array}{c} \mu \left(t\right)=\;{\left[\prod_{i\in \text{Mode}}\text{Mode}\left(i\right)\right]}^{1/\text{Mode}\_\text{Length}}, \end{array}$$

where Mode is the region in the image histogram, Mode(*i*) is the pixels in that region, and Mode_Length is the length of the vector of that mode.

Algorithm 1 shows the single proposed filter experiment when applied on MCET, and for other proposed filter approaches, each filter is to be computed based on their equations, as explained in Section 4 in Algorithm 2.

## 4. Performance Measure

**Figure 5:**
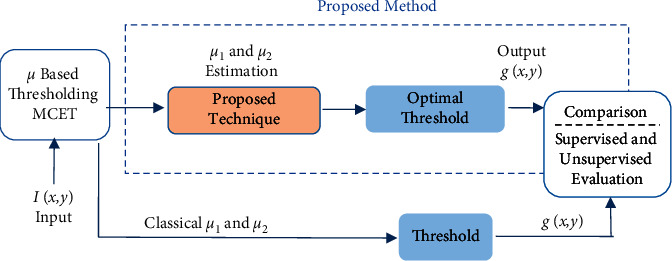
Overall framework of the proposed method and its comparison with the original method.

The segmentation results from the improved minimum cross-entropy thresholding and the original version were compared and evaluated based on the supervised and unsupervised evaluation. These measurements have values that lie between 0 and 1, where the value that is close to 1 refers to a good segmentation result, and vice versa. The overall framework of our proposed method is shown in Figure 5. In unsupervised evaluation, the segmentation results were compared with characteristics of the original image, such as image uniformity, regions contrast, and inter-region disparity. In supervised evaluation, the segmentation results were compared with its references or ground truths images.

### 4.1. Unsupervised Evaluation

Without any a priori information, it is possible to evaluate the quality scores of segmentation results, by using unsupervised evaluation based on the statistics in each region in the segmentation result and its original image. For region segmentation, the various measurement takes into account the uniformity, region contrast, and inter-region disparity.

#### 4.1.1. Image Uniformity

This measurement tends to indicate the quality of the thresholding method, by computing region uniformity based on the variance. This measurement is proposed by Levine et al. [26] and discussed by [8]. The IU is defined in the following equation:

(23) $$\begin{array}{c} IU=1-\frac{\sigma 1^{2}\left(t\right)-\sigma 2^{2}\left(t\right)}{Z}, \end{array}$$

where *σ*1^2^(*t*) and *σ*1^2^(*t*) are the variance of *R*_1_ and *R*_2_, respectively, as shown in Figure 1, and *Z* is calculated as shown in the following equation:

(24) $$\begin{array}{c} Z=\frac{{\left(I_{\max}-I_{\min}\right)}^{2}}{2}, \end{array}$$

where *I*_max_ and *I*_min_ are the minimum and maximum intensity levels, respectively.

#### 4.1.2. Region Contrast

This measurement tends to check the adjacent regions and indicate the high contrast [8]; thus, it evaluates the quality of the segmented image. For a given *t*:

(25) $$\begin{array}{c} RC\left(t\right)=\frac{|\mu_{1}\left(t\right)-\mu_{2}\left(t\right)|}{\mu_{1}\left(t\right)-\mu_{2}\left(t\right)}, \end{array}$$

where *μ*_1_(*t*) and *μ*_2_(*t*) are the estimated mean values of two regions in the image histogram.

#### 4.1.3. Inter-Regions Disparity

This measurement uses the interior contrast and the external contrast in its evaluation [27], as shown in equations (26) and (27), respectively, where *c*(*s*, *t*) is the contrast between two pixels *s* and *t*, as shown in equation (28):

(26) $$\begin{array}{c} CI\left(i\right)=\frac{1}{A_{i}}\sum_{s=R_{i}}\text{Max}{\left\{c\left(s,t\right),t\in W\left(s\right)\cap R\right\}}_{i}, \end{array}$$

(27) $$\begin{array}{c} CE\left(i\right)=\frac{1}{l_{i}}\sum_{s=F_{i}}\text{Max}\left\{c\left(s,t\right),t\in W\left(s\right)\notin R_{i}\right\}, \end{array}$$

(28) $$\begin{array}{c} c\left(s,t\right)=\frac{|I\left(s\right)-I\left(t\right)|}{L-1}, \end{array}$$

where *I*_*i*_ is the length of the border *F*_*i*_ in the region *R*_*i*_, *A*_*i*_ corresponds to the surface of the region *R*_*i*_, and *W* (*s*) is the neighborhood of the pixel *s*.

(29) $$\begin{array}{c} C\left(R_{i}\right)=\left\{\begin{array}{cc} 1-\frac{CI\left(i\right)}{CE\left(i\right)}, & \text{if }0<CI\left(i\right)<CE\left(i\right),\\ CE\left(i\right), & \text{if }CI\left(i\right)=0,\\ \;0, & \text{otherwise.} \end{array}\right. \end{array}$$

The higher value or close to 1 refers to a good segmentation result, while the lower value or close to 0 refers to a poor segmentation result.

### 4.2. Supervised Evaluation

**Figure 6:**
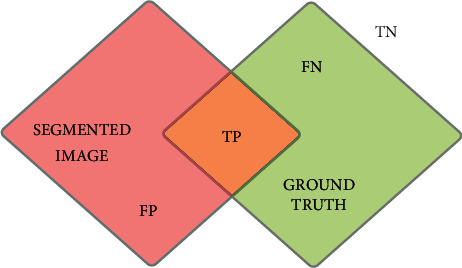
TN represents the pixels that have not been segmented; FN represents the pixels that should appear in the segmented image; TP is the joint segmented pixels; and FP represents the pixels that should not have been segmented.

The comparisons between the segmentation results and its ground truth are a powerful measurement to evaluate segmentation quality scores [28–31]. This measurement is considered a pixels-based evaluation. It is widely used in literature. Given segmentation method *s*, its resulting segment can be written from image pixel set *I* into two disjoint areas *I*=*P*_*s*_ ∪ *N*_*s*_, where *P*_*s*_ and *N*_*s*_ represent the positive and negative pixels, respectively. Similarly for the ground truth *I*=*P*_*g*_ ∪ *N*_*g*_. The goal is to achieve a perfect matching, for example, *P*_*s*_=*P*_*g*_; if this not going to happen, the following sets will be defined as shown in Figure 6:

- True positives (TP): pixels that are segmented but appeared as so in the ground truth: TP=*P*_*s*_∩*P*_*g*_
- False positives (FP): pixels that are segmented but not appeared as so in the ground truth: FP=*P*_*s*_∩*P*_*g*_
- False negatives (FN): pixels that are grouped out of the segmentation but belong to the ground truth: FN=*N*_*s*_∩*P*_*g*_
- True negatives (FN): Pixels that have not been segmented

The goal is to maximize the true positivity of pixels in the segmentation results as shown in Figure 6. Based on these terms, the Jaccard index (JI), *F*-score, and the segmentation accuracy are used to evaluate the good match with the ground truth reference, as shown in the following equations:

(30) $$\begin{array}{c} {\text{Jaccard}}_{\text{index}}=\frac{\text{TP}}{\text{TP}+\text{FP}+\text{FN}}, \end{array}$$

(31) $$\begin{array}{c} F_{\text{Score}}=2*\frac{\text{Precision}*\text{Recall}}{\text{Precision}+\text{Recall}}, \end{array}$$

(32) $$\begin{array}{c} \text{Accuracy}=\frac{\text{TP}+\text{TN}}{\text{FN}+\text{FP}+\text{TP}+\text{TN}}, \end{array}$$

where Jaccard_index_ is to indicate the ratio of intersection, in *F*-score; Precision refers to TP/TP + FP; and Recall refers to TP/TP + FN, where Precision is to measures the detected pixels that are actually true and Recall is to indicate true positivity, and it is the probability that a segmented pixel belongs to the ground truth. Segmentation accuracy is to indicate the degree to which a result from the segmentation algorithm has perfect matches with ground truths.

### 4.3. Modeling the Accurate Segmentation

Different mean filters approaches have been used for MCET to find the best threshold value by minimizing MCET; the various input approaches make the segmentation problems a nondeterministic polynomial (NP) hard optimization problem [6].

Minimizing the value *t* is the process that indicates the optimal threshold *t*^*∗*^ for the image that needs to be accurately segmented; at the same time, the accurate segmentation aims to maximize the evaluation metrics as a better accuracy indicator, as follows:

(33) $$\begin{array}{c} \text{Maximize}\left(\text{Unsupervised,Supervised }\right), \end{array}$$

where unsupervised refers to evaluations (IU(t), RC(t), C(Ri)) and supervised refers to evaluations (Jaccard index, *F*-scores, and Accuracy); they belong to [0,1]. A value close to 0 indicates a poor segmentation result, and a value close to 1 indicates a good segmentation result.

## 5. Performance Evaluation

The proposed method was implemented using MATLAB R2019a 64-bit parallel computing toolbox, with quad-core Intel Core i5, turbo boost up to 3.8 GHz, and 8 GB RAM machine. The proposed algorithm includes five mean filters approaches. In order to consume the computational resources correctly, parallel processing could provide optimal performance, while the sequential process to estimate multiple values could be time-consuming.  Table 3 shows the experimental elapsed time that are recorded to validate the performance of the proposed algorithm.

### 5.1. Modeling Data Sets and Test Cases

Based on the proposed techniques, the segmentation results were obtained using different input parameters for mean estimation with MCET, including the result from the related works, the original MCET that relies on the classical mean, and MCET based on lognormal mean [6]. Three types of medical images were applied (images from OASIS MRI. Alzheimer's disease, MRI brain tumor, and ISIC 2018: skin lesion). These data sets represent various types of image conditions. Nonetheless, each image has been tested with 22 test cases based on mean estimation inputs, as shown in Table 1. This is including the inputs from related works and the proposed mean estimation approach. The average values were computed per test case for each evaluation in the three data sets. A total of 100 images were examined from each data set for unsupervised evaluation with a total of 6,600 test cases, and 50 images were examined from two data sets with references or ground truth for supervised evaluation, with a total of 2,200 test cases. The classical mean in MCET is the original method input. The proposed mean filters are the geometric, harmonic, contraharmonic, and alpha-trimmed filters. Among the proposed approaches, there are 5 selected values of *Q* for the contraharmonic filter, and 13 trim values for the alpha-trimmed filter. These gradual values were selected among a number of experimental test cases based on their positive impact when estimating mean values in each class, where both *μ*_1_ and *μ*_2_ are estimated using one of the proposed mean filters approaches.

### 5.2. Experimental Results and Discussion

The average values of the unsupervised measurement showed various improvement patterns of results, and the ability to detect the fine segmentation structures was achieved, as shown in Tables 1 and 2. MRI Alzheimer's results showed the best performance with alpha trim at *d*/2 = 50. Since the performance decreases gradually when there is an increase in the trim value. Similarly, in the skin lesion images, the trim value at *d*/2 = 55 got the best performance, and so at *d*/2 = 30 for MRI brain tumor images. In the three evaluations, increasing the trim values could positively impact the efficiency or bring it back to its start point of the original method. The three evaluations showed how a change in the mean value could affect the final threshold in MCET. Nonetheless, this change has an important role to improve the segmentation result. This contribution relies on the relation and the dependency between the optimal threshold and the enhanced mean value in the MCET method.

**Figure 7:**
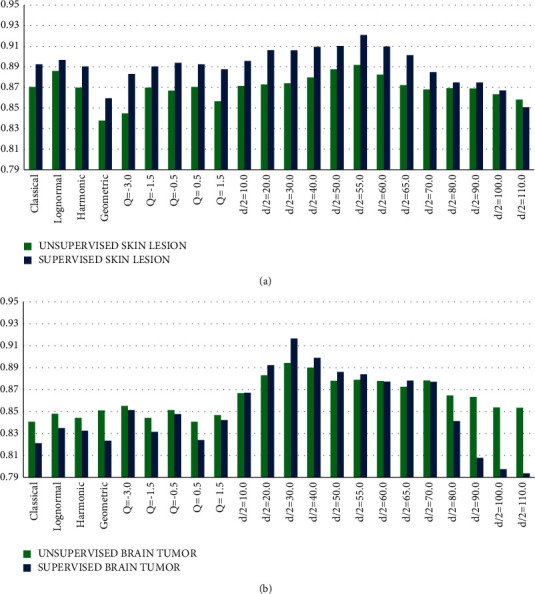
Comparison of evaluation for different mean estimation techniques: (a) unsupervised and supervised evaluation for a skin lesion and (b) unsupervised and supervised for MRI Brain tumors.

In supervised evaluation, skin lesion and MRI brain tumor showed a noticeable evaluation that achieve the improvement goal, as shown in Table 2. Since the supervised evaluations are acting as a matching process with the desired segment or the ground truth. It can be noticed that true positivity has been maximized. Notice that the input parameters in the proposed mean filters, for example, *d*/2 = 0, *Q* = 0, and *Q* = −1, refer to the pre-existed filters; thus, these inputs were ignored to be used. *d*/2 = 0 in the alpha-trimmed filter and *Q* = 0 in contraharmonic filter reduce to the classical mean filter. Also, *Q* = −1 in contraharmonic filter reduces to harmonic filter (Figure 7(a)).

Skin lesion segmentation results showed that both supervised and unsupervised evaluation have the same properties regarding the best result at *d*/2 = 55. They almost have the same pattern of performance, despite the slight changes at some points. The variation between supervised and unsupervised benchmarks is according to the nature of the measurement. Nonetheless, the supervised evaluation is considered more effective for indicating the objectiveness based on the rate of the true positivity in the segmented image, as long as the reference image or the ground truth is available. Similarly, MRI Brain tumor segmentation in Figure 7(b) showed that both unsupervised and supervised evaluation have the best result at *d*/2 = 30 and some identical points at *Q* = −3, *Q* = −0.5, *d*/2 = 10, *d*/2 = 60, and *d*/2 = 70, with noticeable changes in the pattern when larger trims applied with the alpha trim approach.

**Figure 8:**
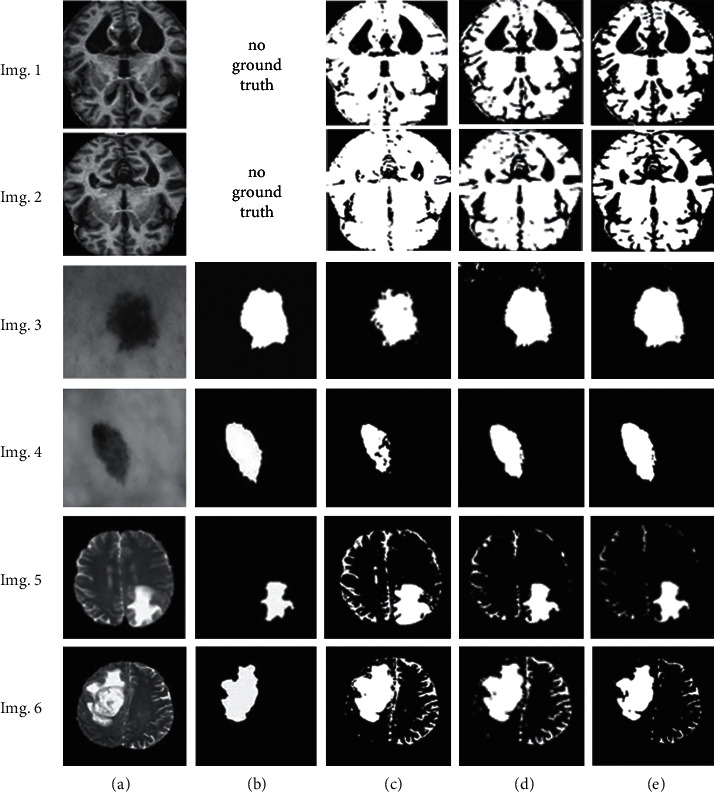
Qualitative illustration for selected samples: (a) original images, (b) ground truths, (c) segmented images using the Gaussian-based MCET, (d) segmented images using the lognormal-based MCET, and (e) segmented images using the proposed MCET, the segmented MRI Alzheimer's Imgs 1.(e) and 2.(e) using alpha trim *d*/2 = 50, the segmented skin lesion Imgs 3.(e) and 4.(e) using alpha trim *d*/2 = 55, and the segmented MRI brain tumor Imgs 5.(e) and 6.(e) using alpha trim *d*/2 = 30.

Figure 8 shows qualitative illustration samples for selected results. MRI Alzheimer's, MRI brain tumor, and dermoscopic skin lesion were segmented using MCET with alpha trim filter for *d*/2 = 50, 55, and 30, respectively. The segmented images appeared with a subjective view, especially when comparing the detected foreground with the ground truth for both MRI brain tumor and skin lesion. However, the objective evaluation scores indicated the segmentation accuracy over MCET-Gaussian distribution, as shown in Table 4.

Segmentation results in the three data sets showed the best improvement when using the mean value from the alpha trim filter with MCET. With the presence of several sizes of images that need to be segmented using this approach, the trim values are submissive to the size of the image. Since there is a positive relationship between the trim values and the size of the tested images, for example, when the image is resized from 128 × 128 to 256 × 256, the trim values should be duplicated.  Table 5 shows the comparison between the proposed MCET method using alpha-trimmed filter with two related methods; the proposed method was able to record a higher increase rate of segmentation accuracy; this is according to the average scores of the unsupervised and the supervised evaluation.

The proposed method recorded a maximum of 4.0% increase rate of accuracy over the original MCET using Gaussian distribution and minimum of 3.6%. Also, it recorded a 1.9% increase rate of accuracy over MCET using lognormal distribution. Knowing that MRI Alzheimer's images were examined using the unsupervised evaluation only for the absence of the ground truth. However, both MRI brain tumor and skin lesions were examined using the supervised and the unsupervised evaluation.

## 6. Conclusion and Future Work

This paper presents an improvement of minimum cross-entropy thresholding (MCET) based on different mean filters approaches. The proposed filtering approaches produced a positive effect on the segmentation performance; this is due to the impact of the enhanced mean value on MCET. The comparison approaches relied on three unsupervised evaluations and three supervised evaluations. The effectiveness of the proposed technique was tested using three types of medical images. This improvement is to contribute to the optimization for images segmentation, to overcome the challenge of fixed threshold value based on the classical mean, and also to provide inclusive usage for thresholding methods for various types of images.

In future work, the aim is to extend the contribution to the wide domain of images segmentation, by applying different data sets, and a larger number of images for each test case, with different input parameters. The proposed work was designed for one threshold that separates bimodal histogram. The same empirical concept could be used for multilevel image thresholding purposes in future work.

## Figures and Tables

**Algorithm 1:**
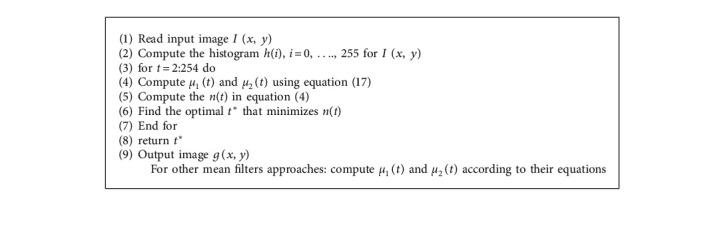
MCET using harmonic mean filters approach.

**Algorithm 2:**
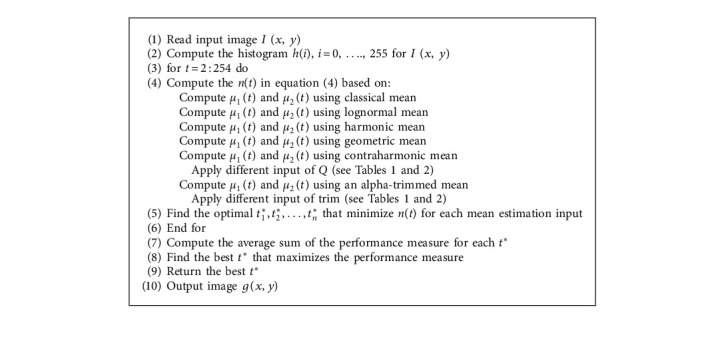
MCET using different mean estimation approaches.

**Table 1:** Average evaluations values of unsupervised performance measurement using the proposed mean estimation techniques compared with the classical mean or Gaussian distribution for MCET.

*μ*_1_, *μ*_2_ estimation			MRI Alzheimer's	Skin lesion	MRI brain
1	Classical mean		0.8841	0.8703	0.8406
2	Lognormal [6]		0.8796	0.8860	0.8479
3	Harmonic		0.8902	0.8697	0.8440
4	Geometric		0.8722	0.8376	0.8513

5	Contraharmonic	*Q* = −3.0	0.8893	0.8446	0.8549
6		*Q* = −1.0	0.8943	0.8697	0.8440
7		*Q* = −0.5	0.8971	0.8669	0.8512
8		*Q* = 0.0	0.8703	0.8702	0.8407
9		*Q* = 0.1	0.8887	0.8603	0.8449

10	Alpha trim	*d*/2 = 10.0	0.8924	0.8714	0.8667
11		*d*/2 = 20.0	0.8971	0.8727	0.8830
12		*d*/2 = 30.0	0.8987	0.8739	**0.8942**
13		*d*/2 = 40.0	0.8973	0.8795	0.8899
14		*d*/2 = 50.0	0.9046	0.8876	0.8779
15		*d*/2 = 55.0	0.8968	0.8917	0.8789
16		*d*/2 = 60.0	0.8922	0.8824	0.8777
17		*d*/2 = 65.0	0.8846	0.8721	0.8724
18		*d*/2 = 70.0	0.8928	0.8679	0.8782
29		*d*/2 = 80.0	0.8842	0.8692	0.8645
20		*d*/2 = 90.0	0.8831	0.8689	0.8633
21		*d*/2 = 100	0.8787	0.8632	0.8536
22		*d*/2 = 110	0.8755	0.8581	0.8534

**Table 2:** Average evaluation values of supervised performance measurement using the proposed mean estimation techniques compared with the classical mean or Gaussian distribution for MCET.

*μ*_1_, *μ*_2_ Estimation			Skin lesion	MRI Brain
1	Classical mean		0.8923	0.8210
2	Lognormal [6]		0.8966	0.8349
3	Harmonic		0.8903	0.8323
4	Geometric		0.8593	0.8233

5	Contraharmonic	*Q* = −3.0	0.8830	0.8513
6		*Q* = −1.0	0.8903	0.8313
7		*Q* = −0.5	0.8938	0.8475
8		*Q* = 0.0	0.8923	0.8240
9		*Q* = 0.1	0.8875	0.8421

10	Alpha trim	*d*/2 = 10.0	0.8957	0.8670
11		*d*/2 = 20.0	0.9060	0.8922
12		*d*/2 = 30.0	0.9059	**0.9160**
13		*d*/2 = 40.0	0.9093	0.8988
14		*d*/2 = 50.0	0.9102	0.8860
15		*d*/2 = 55.0	**0.9208**	0.8839
16		*d*/2 = 60.0	0.9097	0.8772
17		*d*/2 = 65.0	0.9013	0.8781
18		*d*/2 = 70.0	0.8849	0.8771
29		*d*/2 = 80.0	0.8747	0.8412
20		*d*/2 = 90.0	0.8746	0.8077
21		*d*/2 = 100	0.8669	0.7974
22		*d*/2 = 110	0.8507	0.7936

**Table 3:** Time performance of the proposed algorithm when performed using parallel processing.

Segmented images		Sequential (sec)	Parallel (sec)	Speed-up gain (%)
1	MRI Alzheimer's	437.201	278.805	36.23
2	Skin lesion	562.926	317.622	43.58
3	MRI brain	489.003	291.902	40.31

**Table 4:** Comparison of the unsupervised and supervised evaluation for the segmented images in Figure 8.

	Unsupervised evaluation			Supervised evaluation		
	RC	IU	IRD	JI	FS	QLTY
Img.1(c)	0.8709	0.8782	0.8462	—	—	—
Img.1(d)	0.8816	0.8852	0.8398	—	—	—
Img.1(e)	**0.8897**	**0.8989**	**0.8837**	—	—	—
Img.2(c)	0.8630	0.8702	0.8529	—	—	—
Img.2(d)	0.8759	0.8778	0.8408	—	—	—
Img.2(e)	**0.8879**	**0.8899**	**0.8873**	—	—	—
Img.3(c)	0.8519	0.8458	0.8381	0.8379	0.9063	0.9173
Img.3(d)	0.8815	0.8689	0.8473	0.8449	0.9037	0.9290
Img.3(e)	**0.8857**	**0.8939**	**0.8685**	**0.8559**	**0.9131**	**0.9792**
Img.4(c)	0.8498	0.8500	0.8482	0.8398	0.8971	0.9157
Img.4(d)	0.8698	0.8697	0.8688	0.8407	0.9026	0.9238
Img.4(e)	**0.8878**	**0.8941**	**0.8705**	**0.8518**	**0.9099**	**0.9672**
Img.5(c)	0.7509	0.7558	0.7238	0.7210	0.8308	0.7294
Img.5(d)	0.8647	0.8794	0.8600	0.9325	0.9227	0.9371
Img.5(e)	**0.8788**	**0.8899**	**0.8728**	**0.9497**	**0.9549**	**0.9503**
Img.6(c)	0.7618	0.7592	0.7327	0.7292	0.8299	0.7381
Img.6(d)	0.8797	0.8704	0.8599	0.9293	0.9208	0.9305
Img.6(e)	**0.8828**	**0.8878**	**0.8779**	**0.9431**	**0.9459**	**0.9492**

**Table 5:** Overall comparison using average evaluation metrics for the proposed method using the alpha trim approach and two related methods. Row A refers to an unsupervised evaluation, and row B refers to the supervised evaluation.

Original MCET-Gaussian distribution			
Dermoscopic skin lesion		MRI brain tumor	MRI Alzheimer's
A	0.8703	0.8406	0.8841
B	0.8923	0.8210	—

MCET-lognormal distribution			
Dermoscopic skin lesion		MRI brain tumor	MRI Alzheimer's
A	0.8802	0.8693	0.8874
B	0.8817	0.8807	—
Overall increase rate of accuracy over Gaussian = 2.1%			

Proposed MCET-alpha trim *d*/2 = 55			
Dermoscopic skin lesion		MRI brain tumor	MRI Alzheimer's
A	0.8917	0.8789	0.8968
B	0.9208	0.8839	—
Overall increase rate of accuracy over Gaussian = 3.7%			
Overall increase rate of accuracy over lognormal = 1.6%			

Proposed MCET-alpha trim *d*/2 = 50			
Dermoscopic skin lesion		MRI brain tumor	MRI Alzheimer's
A	0.9102	0.8779	0.90426
B	0.8876	0.8860	—
Overall increase rate of accuracy over Gaussian = 3.65%			
Overall increase rate of accuracy over lognormal = 1.5%			

Proposed MCET-alpha trim *d*/2 = 30			
Dermoscopic skin lesion		MRI brain tumor	MRI Alzheimer's
A	0.8739	0.8942	0.8928
B	0.9059	0.9165	—
Overall increase rate of accuracy over Gaussian = 4.0%			
Overall increase rate of accuracy over lognormal = 1.9%			

## Data Availability

Data and materials will be available upon request.
